# Production of Catalyst-Free Hyperpolarised Ethanol Aqueous Solution via Heterogeneous Hydrogenation with Parahydrogen

**DOI:** 10.1038/srep13930

**Published:** 2015-09-09

**Authors:** Oleg G. Salnikov, Kirill V. Kovtunov, Igor V. Koptyug

**Affiliations:** 1 International Tomography Center, SB RAS, 3A Institutskaya St., Novosibirsk, 630090, Russia; 2 Novosibirsk State University, Pirogova St. 2, Novosibirsk, 630090, Russia

## Abstract

An experimental approach for the production of catalyst-free hyperpolarised ethanol solution in water via heterogeneous hydrogenation of vinyl acetate with parahydrogen and the subsequent hydrolysis of ethyl acetate was demonstrated. For an efficient hydrogenation, liquid vinyl acetate was transferred to the gas phase by parahydrogen bubbling and almost completely converted to ethyl acetate with Rh/TiO_2_ catalyst. Subsequent dissolution of ethyl acetate gas in water containing OH^−^ ions led to the formation of catalyst- and organic solvent-free hyperpolarised ethanol and sodium acetate. These results represent the first demonstration of catalyst- and organic solvent-free hyperpolarised ethanol production achieved by heterogeneous hydrogenation of vinyl acetate vapour with parahydrogen and the subsequent ethyl acetate hydrolysis.

Magnetic Resonance Imaging (MRI) is a widely used technique for visualization of various objects, from the routine medical diagnostics of patients to porous media^1^ and catalytic reactors^2^,^3^. However, the capabilities of MRI are largely restricted by its inherently low sensitivity. Therefore, a number of hyperpolarisation techniques^4^,^5^ such as Dynamic Nuclear Polarization (DNP)^6^,^7^, Spin-Exchange Optical Pumping (SEOP)^8^ and Parahydrogen-Induced Polarization (PHIP)^9^,^10^ are often utilized to overcome this problem. DNP is the most frequently used hyperpolarisation technique, but it has significant drawbacks of long polarization cycles (~20–100 min) and quite expensive equipment^4^. In contrast, the PHIP technique allows one to produce hyperpolarised (HP) substances in less than 1 min and is relatively inexpensive^11^. PHIP exploits high spin order of parahydrogen molecule (p-H_2_) which is usually transferred to a substrate of interest via pairwise addition of two H atoms from the same p-H_2_ molecule to a double or a triple bond of the substrate molecule. This requirement of the chemical modification of a substrate significantly limits the range of compounds which can be hyperpolarised by PHIP^5^. This problem can be partially solved by the recently developed version of PHIP technique known as Signal Amplification By Reversible Exchange (SABRE)^12^. SABRE is based on the interactions of p-H_2_ with a suitable ligand upon their reversible binding to a metal complex^13^. In SABRE, the chemical structure of the substrate remains unchanged; however, the catalyst might be changed irreversibly^14^,^15^. Importantly, only a limited range of compounds, mostly nitrogen-containing heterocycles^16^ (e.g. pyridine) and PPh_3_^17^ were hyperpolarised by SABRE so far. The second problem is that both the pairwise hydrogen addition in PHIP and the reversible exchange processes in SABRE are usually executed with the use of homogeneous catalysts such as transition metal complexes, that cannot be easily removed from a reaction mixture^10^,^18^. However, this problem can be solved by using heterogeneous catalysts^19^ such as supported metal nanoparticles^20^,^21^,^22^, immobilized metal complexes^23^ or bulk metals and metal oxides^24^ which are able to produce PHIP effects.

The two problems mentioned above, namely the nature of substrate molecules and the difficulty of separating the hyperpolarised molecules from the reaction mixture, make the preparation of HP contrast agents for biomedical MRI applications using PHIP technique a strongly challenging task because these contrast agents should be biocompatible and absolutely free from toxic catalysts and organic solvents. There are reports about the production of PHIP-hyperpolarised biomolecules such as succinate^25^, phospholactate^26^,^27^, glucose derivatives^28^, peptides^29^ and SABRE-hyperpolarised amino acids, peptides^30^ and drugs^31^. Importantly, all of these results were obtained with the use of homogeneous catalysts which were present in the reaction mixture along with HP molecules, making potential biomedical applications questionable.

An interesting approach which broadens the range of compounds that can be hyperpolarised by PHIP was suggested by Trantzschel *et al.*^32^ They produced HP ethanol, which is not accessible via direct homogeneous hydrogenation, using a two-step procedure consisting of vinyl acetate hydrogenation with p-H_2_ in D_2_O and subsequent alkaline hydrolysis with NaOD solution. However, the problem of separation of homogeneous catalyst present in the reaction mixture along with HP ethanol was not solved in that investigation. The described approach was recently extended by Reineri *et al.*^33^ First, they carried out similar hydrogenation/hydrolysis experiments with vinyl and propargyl acetates with the addition of field cycling procedure between the two chemical reactions which achieved the hyperpolarisation transfer to ^13^C nuclei of carboxylic groups. Thus, ^13^C-hyperpolarised acetate production was implemented. Next, extraction of hyperpolarised molecules from an organic to an aqueous phase was demonstrated for pyruvate. The experimental procedure was similar; however, in this case homogeneous hydrogenation of 2-propynyl-2-oxopropanoate was performed in a mixture of organic solvents (CDCl_3_/CD_3_OD). The addition of an aqueous NaOD solution yielded a biphasic system, and the aqueous phase containing HP pyruvate was used for the NMR investigation. Certainly, this phase extraction procedure allowed the authors to separate the HP product from the homogeneous catalyst; however, the obtained aqueous phase should inevitably contain some amounts of very toxic methanol which is highly undesirable for possible biomedical applications.

The benefit of heterogeneous catalysis is that the catalyst can be easily separated from the reaction mixture even in the case of liquid phase hydrogenation. Moreover, gas phase hydrogenation with parahydrogen allows continuous production of catalyst-free HP substances^11^. Thus, the use of heterogeneous catalysts for the production of biocompatible HP contrast agents (e.g., ethanol) seems to be a very promising approach. However, recent attempts to produce HP alcohols via heterogeneous hydrogenation of C = O bond with parahydrogen were unsuccessful^34^. Therefore, in this communication we present for the first time an approach based on the use of heterogeneous hydrogenation with parahydrogen in combination with hydrolysis for HP ethanol production. It will be demonstrated below that heterogeneous hydrogenation of vinyl acetate with p-H_2_ in the gas phase with subsequent hydrolysis in the liquid phase yields HP ethanol, free from any catalyst and organic solvent. Moreover, our approach enables a continuous production of HP ethanol, which is a significant step toward potential biomedical applications.

## Results and Discussion

It was shown recently that heterogeneous catalysts can be used in liquid phase hydrogenation with parahydrogen to produce polarised contrast agents in the liquid phase^22^. Moreover, utilization of the Rh/TiO_2_ catalyst allows one to achieve pronounced PHIP effects in water^35^. Therefore, we first attempted to apply the same procedure to vinyl acetate hydrogenation by bubbling gaseous mixture of reactants (vinyl acetate(gas) + parahydrogen) into the NMR tube containing D_2_O and the Rh/TiO_2_ catalyst at the bottom (see Supplementary Fig. S2). Indeed, it was found that pronounced PHIP effects can be observed for the CH_3_- and CH_2_-protons of ethyl acetate (see Supplementary Fig. S3). However, because of the very low activity of the catalyst in the heterogeneous liquid phase hydrogenation (confirmed by the absence of any signals of reaction product in the thermal ^1^H NMR spectrum) along with the presence of the catalyst as a solid powder in the reaction mixture, this approach cannot compete with the gas phase hydrogenation which is significantly more efficient in terms of reactant conversion which can reach 100% upon variation of the catalyst amount or the gas flow rate. Therefore, the combination of a very efficient gas phase heterogeneous hydrogenation with the use of the supported rhodium catalyst which favours pairwise route of hydrogen addition leads to the possibility of an efficient use of the PHIP approaches developed previously.

**Figure 1:**
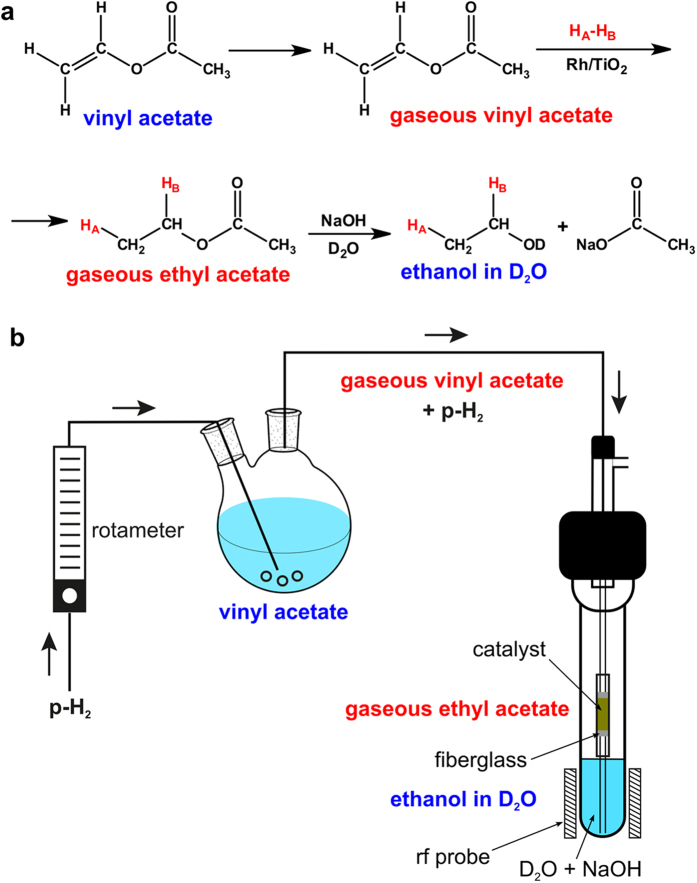
The production of hyperpolarised ethanol. (**a**) The reaction scheme showing its production via heterogeneous gas phase hydrogenation of vinyl acetate with subsequent hydrolysis of the hydrogenation product (ethyl acetate) in aqueous solution, and (**b**) the scheme of experimental setup which was utilized for this purpose. The figure was drawn by O.G. Salnikov.

In our gaseous experiments we used the heterogeneous Rh/TiO_2_ catalyst which was previously shown to yield the strongest HP signals^11^. The experimental setup designed for HP ethanol production is presented schematically in Fig. 1b. The main advantages of this setup are: (i) the spatial separation of the hydrogenation process and the hydrolysis process; (ii) gas phase heterogeneous hydrogenation; (iii) dissolution and hydrolysis in water. This was implemented by placing the catalyst in a PTFE capillary located in the middle part of the NMR tube just above the sensitive zone of the rf probe in order to minimize possible losses of hyperpolarisation intensities caused by relaxation processes during gas transport^36^. The gaseous mixture of reactants obtained by bubbling the parahydrogen through liquid vinyl acetate was supplied through this catalytic reactor (where hydrogenation of vinyl acetate gas to ethyl acetate gas took place) to the bottom of the NMR tube filled with aqueous NaOD solution providing hydrolysis of ethyl acetate to HP ethanol and sodium acetate (see the Methods section for a detailed description of the experiments).

**Figure 2:**
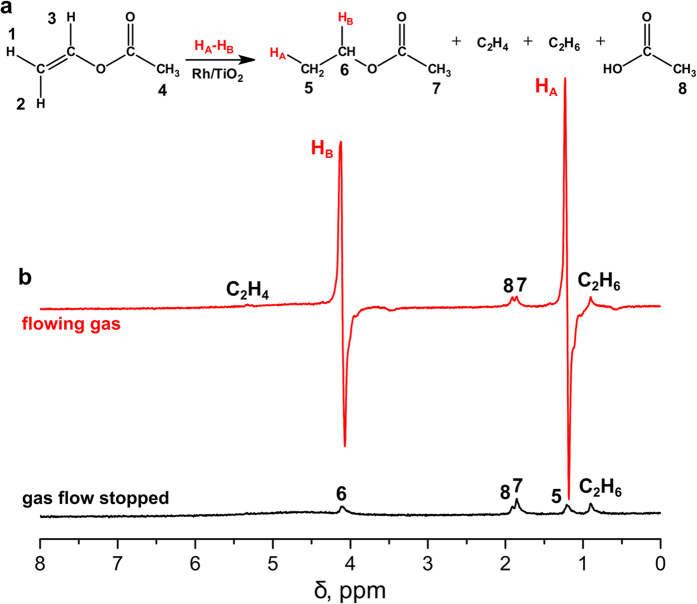
Vinyl acetate hydrogenation in the gas phase. (**a**) The reaction scheme; (**b**) ^1^H NMR spectra acquired in vinyl acetate hydrogenation with parahydrogen with the detection of reaction products in the gas phase under flowing gas conditions (top red trace) and after the termination of gas flow (bottom black trace). The spectra are presented on the same vertical scale.

First of all, the efficiency of pairwise hydrogen addition in heterogeneous hydrogenation of vinyl acetate with p-H_2_ was studied. For this purpose, the hydrolysis step was omitted and the products of the hydrogenation reaction were detected in the gas phase using ^1^H NMR spectroscopy. Because hydrogen addition occurred in the strong magnetic field of the 7 T NMR spectrometer, the antiphase PASADENA^37^ patterns should be expected. The acquired ^1^H NMR spectra indeed contained the signals of CH_3_- and CH_2_-protons of ethyl acetate which clearly exhibited PASADENA effects with the enhancement factors not less than 15 and 20, respectively (Fig. 2). Importantly, the observed NMR spectra did not contain any signals of the reactant (vinyl acetate) which implies that reactant conversion amounted to at least 95% (according to signal-to-noise ratio measurements).

Next, we aimed to figure out whether the hyperpolarisation of ethyl acetate can be preserved upon its dissolution in D_2_O. For this purpose, the experimental setup presented in Fig. 1 was used except that NMR tube was filled with pure D_2_O instead of the NaOD solution. The ^1^H NMR spectra revealed that PASADENA hyperpolarisation was indeed preserved to some extent upon dissolution of ethyl acetate in D_2_O (see Supplementary Fig. S4). This situation is similar to the previously reported results on the dissolution of hyperpolarised gases with the preservation of significant level of polarization produced by PHIP^38^.

**Figure 3:**
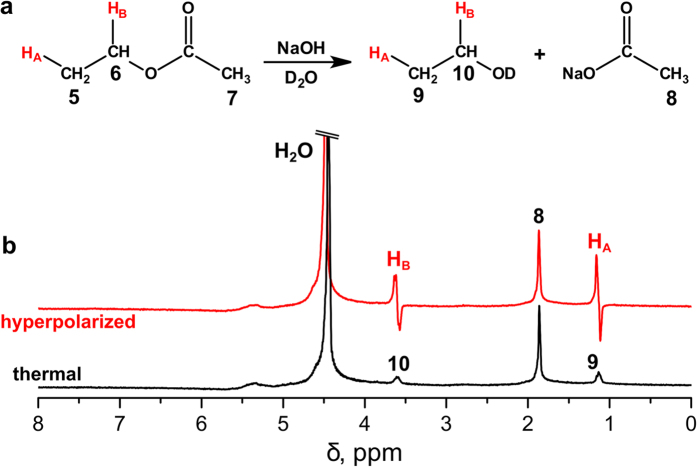
Hydrolysis of hyperpolarised ethyl acetate. (**a**) The reaction scheme; (**b**) ^1^H NMR spectra acquired in vinyl acetate hydrogenation with parahydrogen with subsequent hydrolysis of the product in 1 M NaOD solution immediately after the gas flow was stopped (red line) and a few seconds later after the complete relaxation of polarization (black line). The spectra are presented on the same vertical scale.

Generally, for the HP ethanol formation the hydrolysis of hyperpolarised ethyl acetate should be performed. Therefore, the final step was to combine the hydrogenation and hydrolysis procedures. This was implemented by filling the NMR tube with 2 mL of 1 M NaOD solution in D_2_O. The acquired ^1^H NMR spectra clearly show the antiphase PASADENA signals of hyperpolarised ethanol (Fig. 3). Importantly, this is the first presentation of the technique for the HP ethanol production by combing the gas phase heterogeneous hydrogenation and the liquid phase hydrolysis of the product. In addition, it is the first observation of PASADENA type ^1^H NMR spectra of HP ethanol; previously only the ALTADENA^39^ type effects for HP ethanol were reported^32^.

It should be noted that signals of ethyl acetate and vinyl acetate were not observed in the NMR spectra detected after hydrolysis, implying that the hydrolysis step proceeded quantitatively. This was further confirmed by vinyl acetate hydrolysis experiments. The procedure was the same as described earlier except the heterogeneous catalyst was removed from the gas flow path. In the acquired ^1^H NMR spectrum, only the signals of acetate ion and the residual protons of D_2_O were observed, demonstrating the complete hydrolysis of vinyl acetate (see Supplementary Fig. S5). The absence of acetaldehyde NMR signals may be explained by oligomerisation of acetaldehyde via aldol condensation, which is known to occur in alkaline media^40^. This assumption is confirmed by the fact that the solution progressively became yellow, and orange precipitate was formed, which can be easily explained by the formation of oligomers with several conjugated C = C bonds (see Supplementary Fig. S5). We note, however, that the formation of acetaldehyde and aldol condensation products and the associated colour change of the solution are caused by the omission of the hydrogenation step in this control experiment, but are not present when the hydrogenation/hydrolysis combination is used for ethanol production.

In addition to ethanol, ethyl acetate hydrolysis also leads to the formation of sodium acetate from the carboxylic moiety. We note, however, that this is not a problem for *in vivo* applications because acetate is an endogenous substance metabolized, e.g., via the Krebs cycle^41^. In fact, production of ethyl acetate can be advantageous. Indeed, the ^13^C-hyperpolarised acetate produced by DNP was previously utilized for *in vivo* studies of the TCA cycle^42^.

In conclusion, the presented approach based on heterogeneous hydrogenation of vinyl acetate and hydrolysis of the hydrogenation product ethyl acetate enables the production of catalyst-free HP ethanol by means of the PHIP technique. The use of heterogeneous Rh/TiO_2_ catalyst allows one to perform a very efficient hydrogenation in the gas phase, which has several important advantages compared to liquid phase hydrogenation for the production of HP contrast agents for potential biomedical applications. First of all, the HP reaction product and the catalyst reside in different phases and are separated without any effort. Second, no organic solvent is needed in order to make hydrogenation process efficient. Third, high conversion of the substrate can be achieved even at relatively high gas flow rates. Fourth, HP ethanol can be produced continuously, and no additional steps for catalyst removal are required including decantation of the hyperpolarised solution. Thus, we demonstrated for the first time that HP ethanol which is free from catalyst and organic solvents can be efficiently and continuously produced using heterogeneous hydrogenation in the gas phase with subsequent hydrolysis of the hydrogenation product in aqueous phase. We believe that this approach is very promising for potential applications in medicine, especially when combined with hyperpolarisation transfer to ^13^C nuclei^43^ and/or with the unique properties of long-lived spin states^44^ to significantly prolong the hyperpolarisation lifetime. Importantly, utilisation of ^13^C hyperpolarized ethanol to monitor the oxidation of ethanol to acetate *in vivo* in real time was reported recently^43^. By applying the procedure presented by Reineri *et al.*^33^ it should be possible to neutralize the excess of NaOD with aqueous DCl solution and, importantly, to obtain both highly polarised ethanol and sodium acetate which are free from any catalyst and organic solvent.

## Methods

Commercially available chemicals vinyl acetate (Sigma-Aldrich, >99%), hydrogen, sodium hydroxide (Fluka, >98%) and D_2_O were used as received. Rh/TiO_2_ catalyst (1 wt. % Rh, 1.7 nm mean particle size) was provided by the group of Prof. V.I. Bukhtiyarov (Boreskov Institute of Catalysis, Novosibirsk, Russia) and was described elsewhere^11^. For PHIP experiments, H_2_ gas was enriched with parahydrogen up to 50% by passing it through FeO(OH) powder (Sigma-Aldrich) maintained at liquid nitrogen temperature. The obtained H_2_ gas with 1:1 ortho/para ratio was collected in a gas cylinder.

Hydrogenation experiments were performed as follows. Parahydrogen-enriched H_2_ gas was bubbled through a two-neck flask containing liquid vinyl acetate^34^. The resulting mixture of H_2_ gas and vinyl acetate vapour was preheated to 150 °C by passing it through a 1/8” outside diameter copper tube (~2.5 m in length) folded several times and placed in the Nabertherm tube furnace. Then the gas mixture was supplied through a 1/16” PTFE capillary to a 10 mm NMR tube located in the NMR spectrometer and maintained at 90 °C. In vinyl acetate hydrolysis experiments, the gas mixture was supplied directly to the bottom of the NMR tube containing 2 mL of 1 M NaOH solution in D_2_O. In all other experiments, vinyl acetate hydrogenation was performed. For that purpose the Rh/TiO_2_ catalyst (16.0 mg) was placed in a 1/8” PTFE capillary between the two pieces of fibreglass tissue. This reactor was located in the middle part of the NMR tube, so that hydrogenation occurred in the high magnetic field of the NMR spectrometer (the PASADENA experiment)^37^. The NMR tube part of the experimental setup is schematically presented in Supplementary Fig. S1. Note that the reactor was located as close as possible to the spectrometer sensitive zone in order to minimize the travel time and the corresponding hyperpolarisation losses. The gas mixture passed through the reactor and then to the bottom of the NMR tube. The tube was empty in the case of gas phase HP ethyl acetate detection, contained 2 mL of pure D_2_O in the case of HP ethyl acetate dissolution experiments, or contained 2 mL of 1 M NaOD solution in the case of HP ethyl acetate hydrolysis experiments. The gas exited through the 1/4” PTFE tube connected to the NMR tube at the top. All experiments were carried out at atmospheric pressure. The gas flow rate was set to 14.0 mL/s with the use of an Aalborg rotameter.

^1^H NMR spectra were acquired using a single π/4 rf pulse which provides the maximum PASADENA signal intensities. The spectra were acquired in a single scan on a 300 MHz Bruker AV 300 NMR spectrometer. The NMR spectra of gaseous mixtures were acquired without interrupting gas flow. The NMR spectra of liquid solutions were acquired after a rapid interruption of gas flow in order to avoid magnetic field inhomogeneities caused by bubbles. The NMR spectra of liquids and gases in thermal equilibrium were acquired after the complete relaxation of hyperpolarisation with the stopped gas flow.

## Additional Information

**How to cite this article**: Salnikov, O. G. *et al.* Production of Catalyst-Free Hyperpolarised Ethanol Aqueous Solution via Heterogeneous Hydrogenation with Parahydrogen. *Sci. Rep.* **5**, 13930; doi: 10.1038/srep13930 (2015).

## Supplementary Material

Supplementary figures
