# Supplementary material for: Production of Catalyst-Free Hyperpolarised Ethanol Aqueous Solution via Heterogeneous Hydrogenation with Parahydrogen
Source: Sci Rep. 2015 Sep 9;5:13930. doi: 10.1038/srep13930 (PMC4642547; doi:10.1038/srep13930)
Supplement: Supplementary figures [file srep13930-s1.pdf]

Production of Catalyst-Free Hyperpolarised Ethanol Aqueous Solution via Heterogeneous Hydrogenation with Parahydrogen

Oleg G. Salnikov[1,2], Kirill V. Kovtunov[1,2], and Igor V. Koptyug[1,2,*]

[1]*International Tomography Center, SB RAS, 3A Institutskaya St., Novosibirsk, 630090, Russia*

[2]*Novosibirsk State University, Pirogova St. 2, Novosibirsk, 630090, Russia*

**koptyug@tomo.nsc.ru*

Supplementary Figures

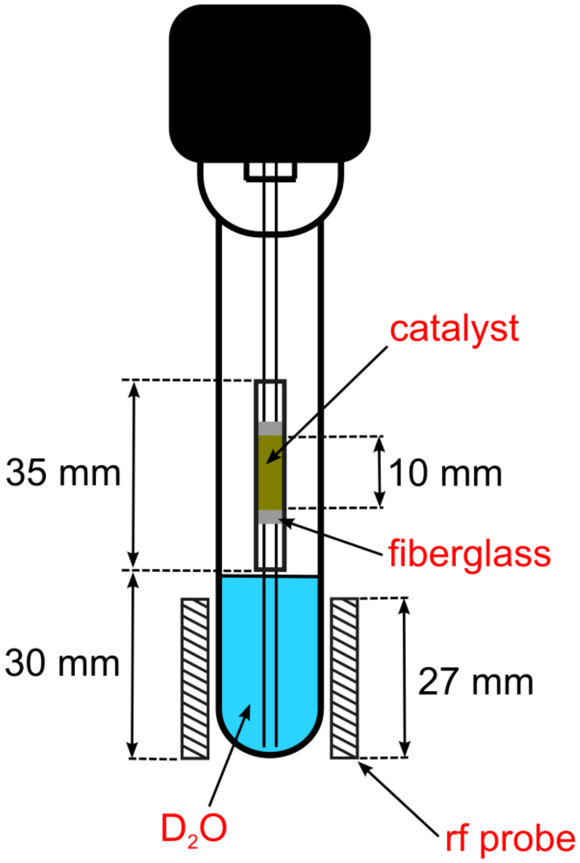

Supplementary Figure S1. The scheme of the NMR tube part of the experimental setup in the case of HP acetate dissolution or hydrolysis experiments. In other experiments, either D_2O or the reactor with the catalyst were removed (see main text for details). The figure was drawn by O.G. Salnikov.

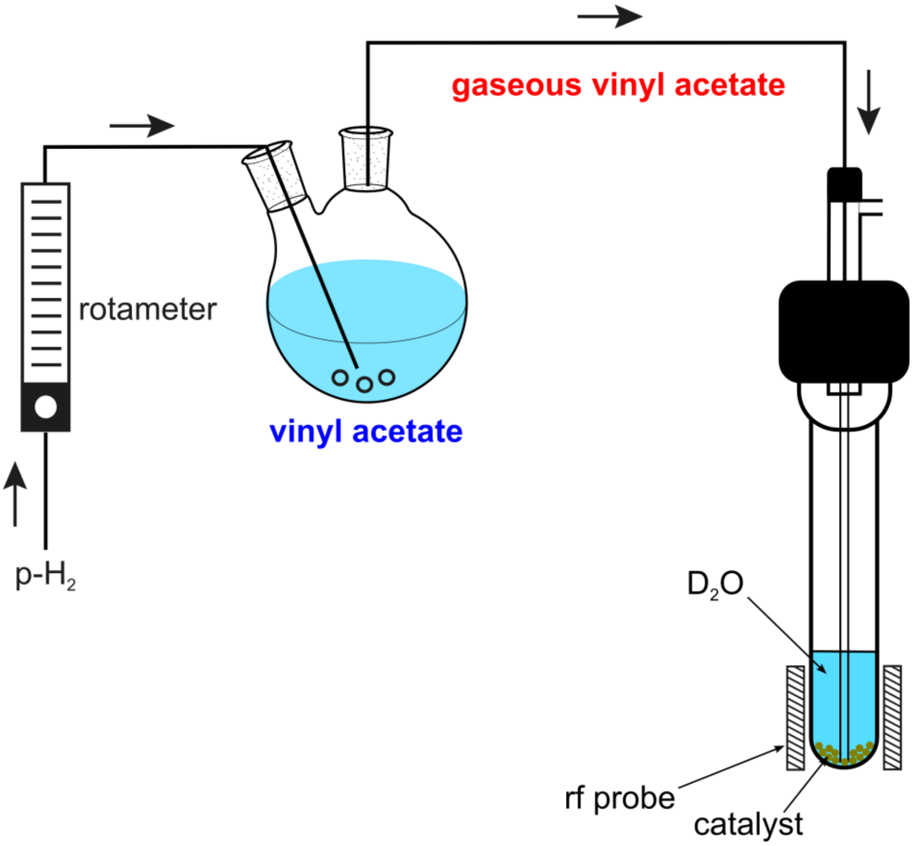

Supplementary Figure S2. The scheme of the experimental setup which was used for liquid phase hydrogenation of vinyl acetate. The figure was drawn by O.G. Salnikov.

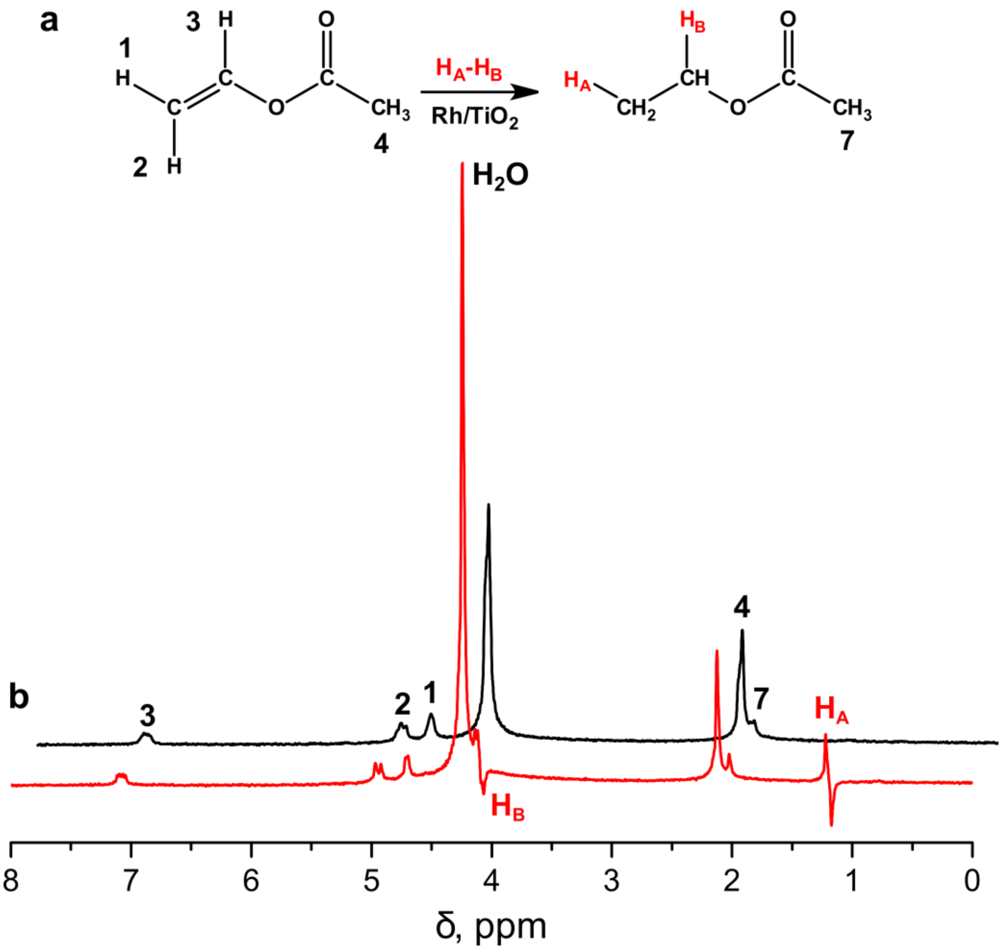

Supplementary Figure S3. (a) The reaction scheme for vinyl acetate hydrogenation in the liquid phase; (b) the ^{1}H NMR spectra acquired in liquid phase vinyl acetate hydrogenation with parahydrogen in D_2O immediately after the gas flow was stopped (red line) and a few seconds later after the complete relaxation of hyperpolarisation (black line).

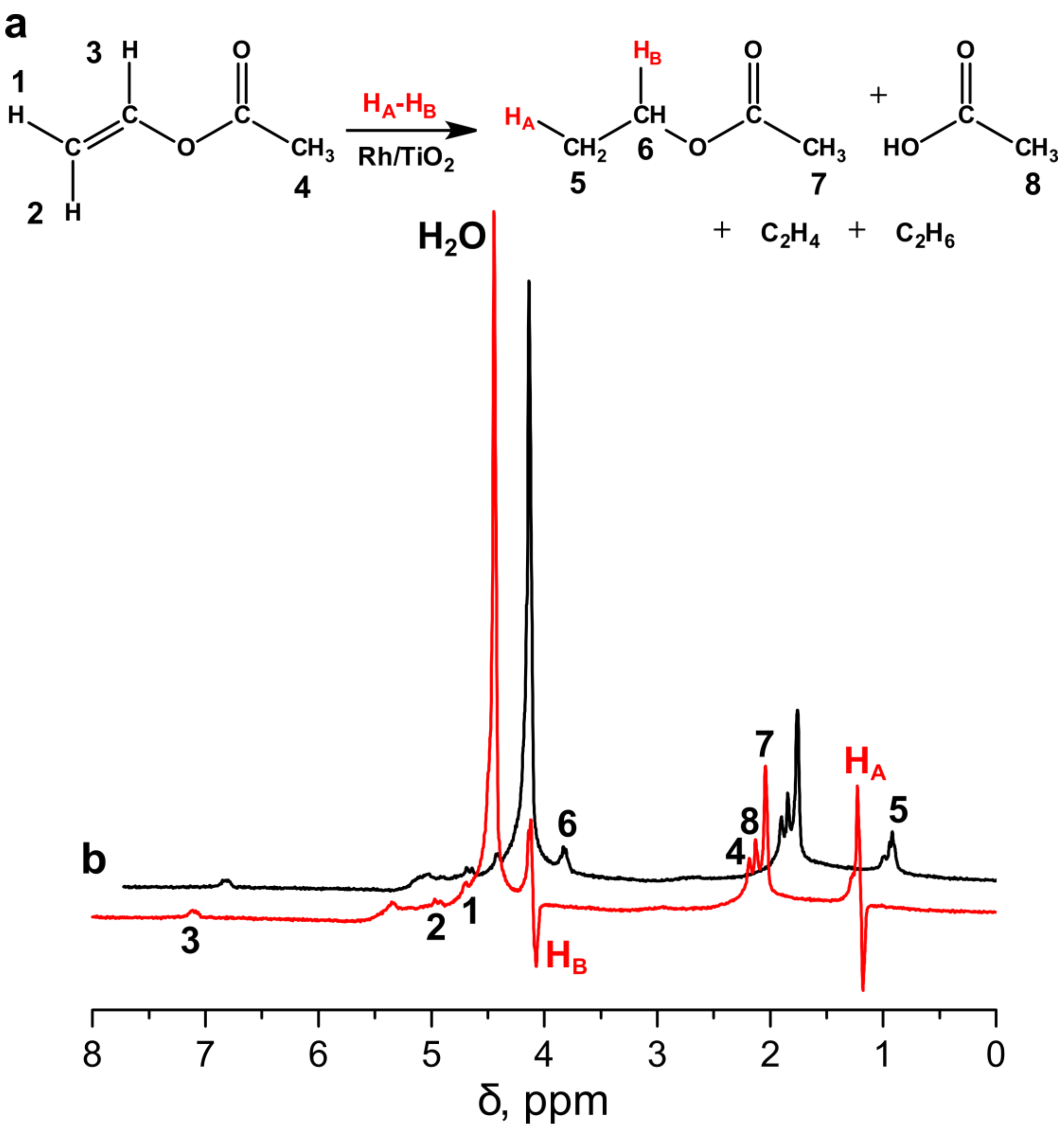

Supplementary Figure S4. (a)The reaction scheme for vinyl acetate hydrogenation in the gas phase; (b) ^{1}H NMR spectra for vinyl acetate hydrogenation with parahydrogen with subsequent dissolution in D_2O acquired immediately after the gas flow was stopped (red line) and a few seconds later after the complete relaxation of hyperpolarisation (black line).

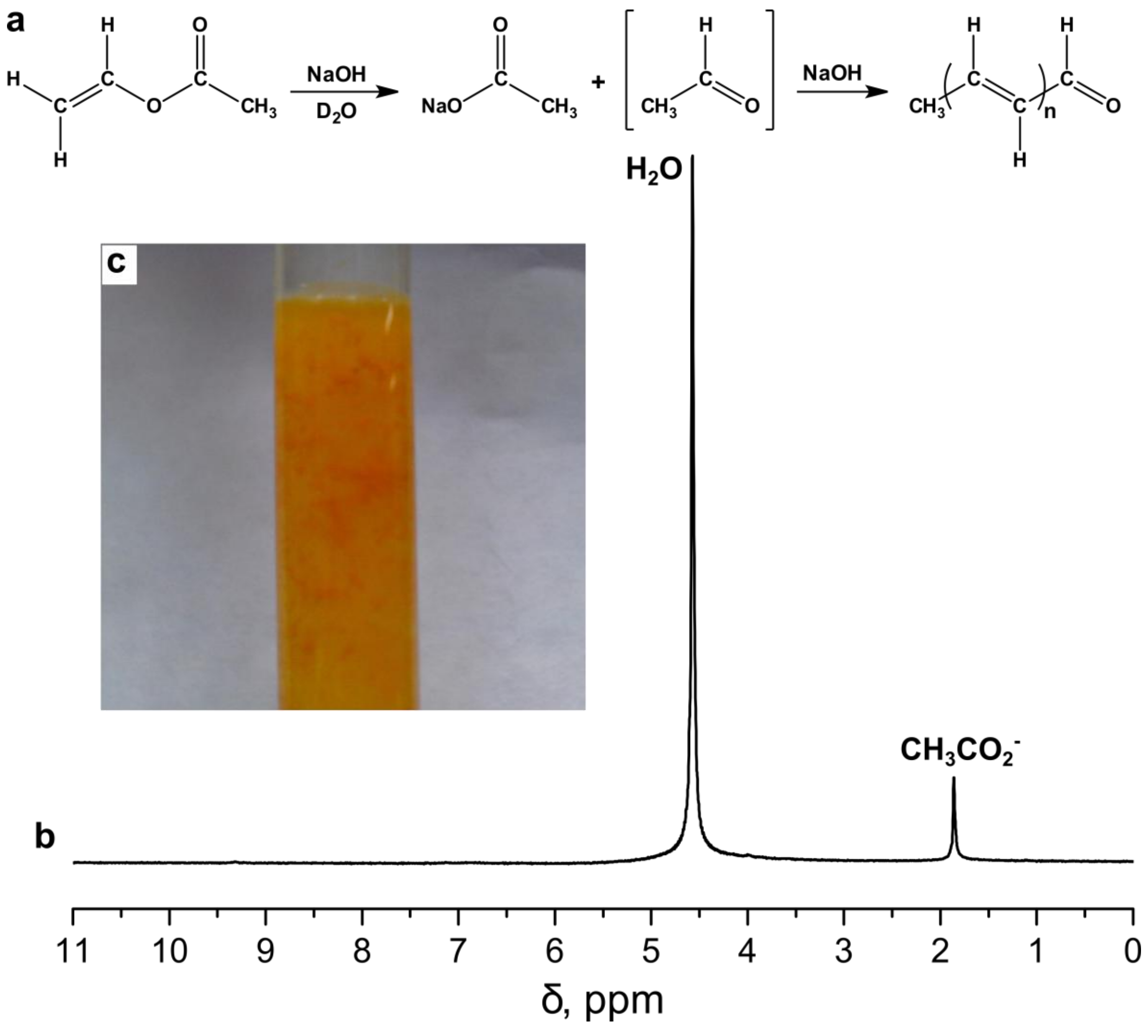

Supplementary Figure S5. (a) The reaction scheme for vinyl acetate hydrolysis; (b) the ^{1}H NMR spectrum acquired upon vinyl acetate hydrolysis in 1 M NaOD solution; (c) the photograph of the NaOD solution after the bubbling of gaseous vinyl acetate/H_2 mixture.
